# Efficacy and safety of secukinumab in patients with psoriatic arthritis: A meta-analysis of different dosing regimens

**DOI:** 10.6061/clinics/2021/e2820

**Published:** 2021-09-20

**Authors:** Kai-Lin Zhang, Si-Yuan Hou, Dan Wu

**Affiliations:** I China Medical University - The Queen’s University of Belfast Joint College, Shenbei New District, Shenyang, Liaoning 110122, China.; II Intensive Care Unit, The People’s Hospital of Liaoning province, Shenhe District, Shenyang, Liaoning 110016, China.; III Second Department of Rheumatology, Shengjing Hospital of China Medical University, Tiexi District, Shenyang, Liaoning 110022, China.

**Keywords:** Secukinumab, Psoriatic Arthritis, Meta-Analysis, Dosing Regimens

## Abstract

The appropriate dosing regimens of secukinumab for psoriatic arthritis (PsA) are not well defined. We performed a meta-analysis to evaluate the efficacy and safety of different dosing regimens of secukinumab in the treatment of PsA.

A systematic search was conducted using major electronic databases to identify relevant randomized controlled trials (RCTs) comparing secukinumab 300 mg *versus* secukinumab 150 mg in patients with PsA. Meta-analysis was performed using Review Manager software (version 5.3).

Six studies with a total of 1141 patients were included. At week 24, secukinumab 300 mg was associated with a higher American College of Rheumatology 20% response (ACR 20), ACR 50, PASI 75 response rate, and dactylitis resolution rate than secukinumab 150 mg, especially in the anti-TNF-IR subgroup. At week 52, secukinumab 300 mg was associated with a higher psoriasis area and severity index (PASI) 75 and PASI 90 response rate than secukinumab 150 mg. There was no significant difference between secukinumab 300 mg and secukinumab 150 mg in the risk of any adverse events (AEs) and serious AEs at either week 24 or week 52.

Secukinumab 300 mg was significantly more effective than 150 mg, especially for patients with PsA who have failed TNF therapy, and it was well tolerated.

## INTRODUCTION

Psoriatic arthritis (PsA) is an immune-mediated chronic inflammatory disease characterized by peripheral arthritis, axial disease, dactylitis, enthesitis, and skin psoriasis, and is associated with impaired physical function and poor quality of life (1). Many patients manifest irreversible joint damage and disability as the disease progresses (2). Indeed, approximately half of the patients manifest bone erosion within 2 years (3). In PsA, structural damage, especially joint space narrowing, affects body function. The irreversible component of impaired body function is strongly related to the extent of joint destruction (4). To maximize the health-related quality of life of patients with PsA, preventing structural damage has become a key treatment goal (5).

Tumor necrosis factor (TNF) is recognized as a treatment for PsA, which significantly improves outcomes in patients with PsA (6). Many patients experience inadequate disease control, treatment intolerance, or insufficient response over time (7). An enhanced understanding of PsA pathogenesis has contributed to the development of targeted therapies. Interleukin 17A (IL-17A) and its receptors are expressed in synovial tissues and can mediate a variety of effector functions. These functions can result in joint inflammation and tissue damage and remodeling (8). Therefore, the IL-17A pathway has been proposed to play a key role in PsA pathogenesis (9-11).

Secukinumab, a human monoclonal antibody that directly inhibits IL-17A, has shown efficacy in treating immune-mediated inflammatory diseases such as psoriasis (7,12), ankylosing spondylitis (AS) (13-15), and rheumatoid arthritis (RA) (16-18). In patients with PsA, secukinumab has been shown to significantly and sustainably improve long-term efficacy and inhibit radiographic progression with a consistent safety profile in several randomized, multicenter, double-blind, placebo-controlled studies (19-24). At present, 300, 150, and 75 mg are the most common doses of secukinumab used, and all doses exhibit significant efficacy (compared with placebo) for treating PsA. Secukinumab has been approved for the treatment of active PsA in Europe since 2015 (5). However, a limitation of the current study is that the appropriate dosing regimens of secukinumab for PsA are not well defined. Therefore, a meta-analysis was conducted to provide an up-to-date and comprehensive conclusion on the efficacy and safety of different dosing regimens of secukinumab for patients with PsA.

## METHODS

### Eligibility criteria

Studies included in this meta-analysis met the following criteria: (i) they were randomized controlled trials (RCTs) that enrolled patients with PsA; (ii) duration of treatment as the main limitation was 24 weeks and the secondary limitation was 52 weeks; (iii) they used a parallel design or crossover design of secukinumab 300 mg *versus* secukinumab 150 mg; and (iv) reported data regarding the American College of Rheumatology (ACR) response, psoriasis area and severity index (PASI) response, dactylitis resolution, enthesitis resolution, and adverse events (AEs).

### Search strategy

We searched all relevant studies published in PubMed, Embase, Web of Science, and Cochrane Library from the time of inception of each database until August 2020, using the following search terms: ‘psoriatic arthritis’ and ‘secukinumab.’ Additionally, the Center Watch Clinical Trials Listing Service (http://www.centerwatch.com), Current Controlled Trials Service (http://www.controlled-trials.com), and clinical trials registered at ClinicalTrials.gov (http://clinicaltrials.gov) were searched for details of any relevant clinical trials in progress.

### Data extraction

Study selection was performed by two independent investigators. They reviewed the full papers to confirm that all trials met the eligibility criteria. Discrepancies were resolved through discussion or by consensus with a third author. When there were multiple studies from the same trial, the reported data that met our evaluation indicators and observation times were eligible.

### Methodological quality

The methodological quality of the included articles was further assessed using modified Jadad criteria with an 8-item scale (randomization, method of randomization, blinding, method of blinding, withdrawals and dropouts, inclusion and exclusion criteria, adverse effects, and statistical analysis) by two independent reviewers (25). Scores ranged from 0 to 8 (a high score indicating high quality), with a score of ≥4 indicating high quality.

### Meta-analysis

Efficacy analysis was based on the proportion of patients with ACR20, ACR50, ACR70, PASI 75, and PASI 90 responses. Additionally, the resolution of enthesitis and dactylitis in the population from baseline was analyzed to assess efficacy. Safety was evaluated by reviewing AEs, including any AEs, serious AEs (SAEs), and Candida infections.

In order to assess the potential confounding effects of heterogeneity, we divided patients who were TNF inhibitor naive (anti-TNF-naive) and those who exhibited inadequate response to TNF inhibitors (anti-TNF-IR) before enrollment into different subgroups.

Statistical analysis was performed using Review Manager 5.3 (The Nordic Cochrane Center, Copenhagen, Denmark) from the Cochrane Collaboration. All analysis indicators, which were categorical dichotomous variables, were assessed using odds ratios (ORs). Statistical significance was set at *p*<0.05, and a 95% confidence interval (CI) was reported. Homogeneity was detected using I^2^ statistics. If the I^2^ statistic was significant (I^2^>50%), a random-effects model was employed; otherwise, a fixed-effects model was used.

## RESULTS

### Literature search and study characteristics

**Figure 1:**
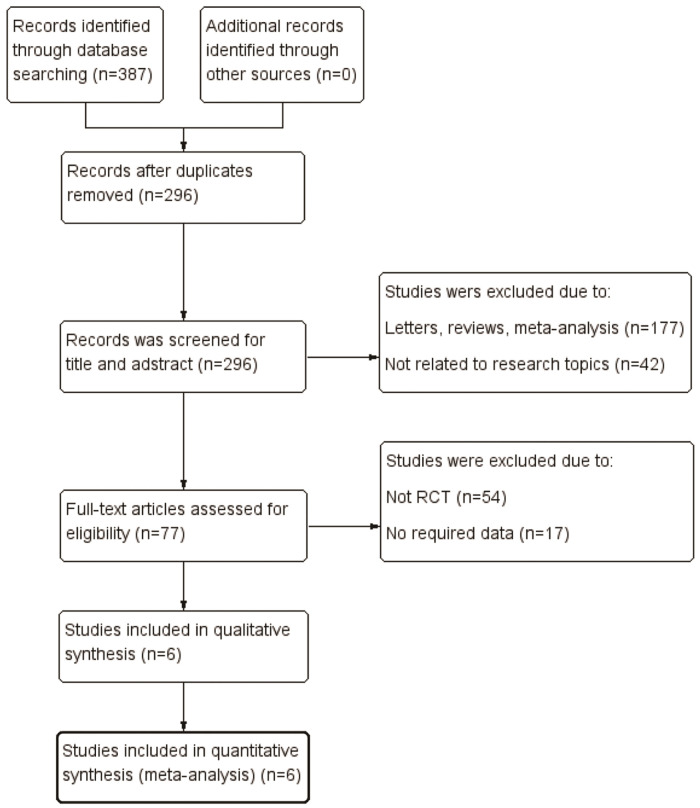
Flow diagram depicting the study selection process.

We identified 387 relevant articles from various electronic databases up to August 31, 2020. After removing duplicates, 296 studies were retrieved. After reviewing the titles and abstracts, 219 articles were excluded, and 77 articles were assessed for eligibility. However, 70 of these were excluded for various reasons, such as not being an RCT, no required data, or no use of secukinumab 300 mg and 150 mg doses. Finally, six studies (19-24) including three RCTs met the eligibility criteria in the final analysis (Figure 1). All three pivotal trials (FUTURE 2, FUTURE 3, and FUTURE 5) were multicenter, double-blind, and placebo-controlled trials with two different secukinumab doses (300 mg and 150 mg). Five articles (19-23) reported the efficacy and safety of secukinumab 300 mg *versus* secukinumab 150 mg during the induction treatment period (24 weeks). Four articles (20-22,24) reported the efficacy and safety of secukinumab 300 mg *versus* secukinumab 150 mg during the maintenance treatment period (≥52 weeks). The pooled analysis included 1141 patients with PsA (461 in the secukinumab 300 mg group and 680 in the secukinumab 150 mg group). All included studies were allocated high-quality scores (modified Jadad score=8). The main study characteristics are presented in Table 1.

### Efficacy

#### ACR20 response

**Figure 2:**
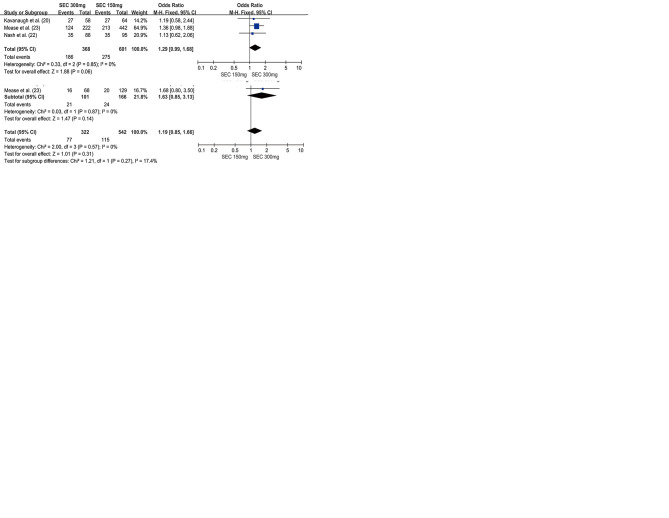
Forest plot of the efficacy between secukinumab 300 mg and secukinumab 150 mg for psoriatic arthritis at week 24.

Three RCTs with 1040 patients reported the proportion of patients meeting the ACR20 improvement criteria at week 24. The secukinumab 300 mg group was associated with a higher ACR 20 response rate than the secukinumab 150 mg group (OR=1.41, 95% CI=1.09-1.83, *p*=0.010). Subgroup analysis revealed that the secukinumab 300 mg group was associated with higher ACR20 responders than the secukinumab 150 mg group in the anti-TNF-IR subgroup at week 24 (OR=1.75, 95% CI=1.13-2.71, *p*=0.01). However, in the anti-TNF-naive subgroup, there was no significant difference between secukinumab 300 mg and secukinumab 150 mg with respect to achieving an ACR20 response at week 24 (OR=1.26, 95% CI=0.91-1.74, *p*=0.17) (Figure 2a).

**Figure 3:**
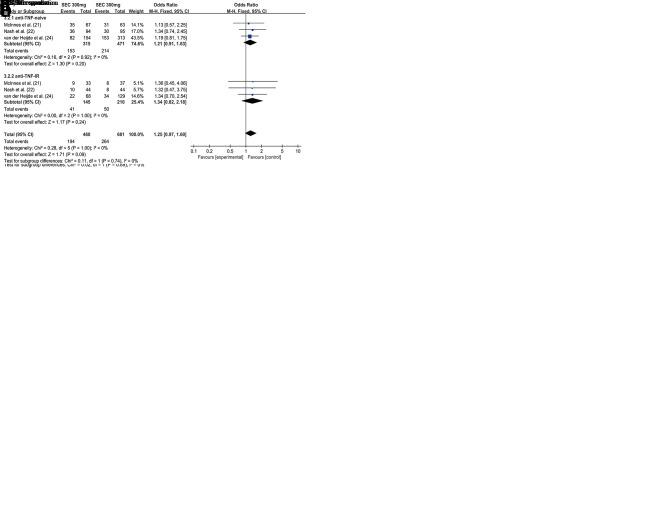
Forest plot depicting the efficacy of secukinumab 300 mg and secukinumab 150 mg in the treatment of patients with psoriatic arthritis at week 52.

Three RCTs with 1141 patients evaluated the ACR20 response rate at week 52. There was no significant difference between the secukinumab 300 mg group and secukinumab 150 mg group with respect to achieving ACR20 response (OR=1.26, 95% CI=0.97-1.62, *p*=0.08) at week 52. However, there were different results in the subgroup analysis. The secukinumab 300 mg group was associated with higher ACR20 responders than the secukinumab 150 mg group in the anti-TNF-IR subgroup at week 52 (OR=1.66, 95% CI=1.07-2.58, *p*=0.01). In the anti-TNF-naive subgroup, there was no significant difference between secukinumab 300 mg and secukinumab 150 mg with respect to achieving an ACR20 response at week 52 (OR=1.08, 95% CI=0.79-1.49, *p*=0.17) (Figure 3a).

#### ACR 50 response

RCTs with 1040 patients reported the proportion of patients meeting the ACR 50 improvement criteria at week 24. The secukinumab 300 mg group was associated with a higher ACR 50 response rate than the secukinumab 150 mg group (OR=1.34, 95% CI=1.02-1.75, *p*=0.03). The proportion of ACR 50 responders was statistically higher in the secukinumab 300 mg group than that in the secukinumab 150 mg group, especially in the anti-TNF-IR subgroup (OR=1.85, 95% CI=1.11-3.08, *p*=0.02). However, in the anti-TNF-naive subgroup, there was no significant difference between secukinumab 300 mg and secukinumab 150 mg with respect to achieving an ACR 50 response (OR=1.18, 95% CI=0.86-1.62, *p*=0.30) (Figure 2b).

Three RCTs with 1141 patients evaluated the ACR50 response rate at week 52. There was no significant difference between secukinumab 300 mg and secukinumab 150 mg with respect to achieving an ACR 50 response, for both the anti-TNF-naive (OR=1.21, 95% CI=0.91-1.63, *p*=0.20) or anti-TNF-IR subgroup (OR=1.34, 95% CI=0.82-2.18, *p*=0.24) (Figure 3b).

#### ACR 70 response

Two RCTs with 864 patients reported the proportion of patients meeting the ACR 70 improvement criteria at weeks 24 and 52, respectively. There was no significant difference between the secukinumab 300 mg group and the secukinumab 150 mg group with respect to achieving an ACR 70 response for both the anti-TNF-naive (OR=1.06, 95% CI=0.72-1.57, *p*=0.75) and anti-TNF-IR subgroup (OR=1.63, 95% CI=0.85-3.13, *p*=0.14) at week 24 (Figure 2c). There was also no significant difference with respect to achieving an ACR 70 response at week 52 for both the anti-TNF-naive (OR=1.23, 95% CI=0.85-1.78, *p*=0.27) and anti-TNF-IR subgroup (OR=1.17, 95% CI= 0.61-2.23, *p*=0.64) (Figure 3c).

#### PASI 75 response

Three RCTs with 893 patients reported the proportion of patients meeting the PASI 75 improvement criteria at week 24. Secukinumab 300 mg was associated with a higher PASI 75 response rate than secukinumab 150 mg (OR=1.49, 95% CI=1.12-1.99, *p*=0.006) (Figure 2d).

Three RCTs with 464 patients evaluated the PASI 75 response rate at week 52. Secukinumab 300 mg was also associated with a higher PASI 75 response rate than secukinumab 150 mg (OR=1.83, 95% CI=1.23-2.73, *p*=0.003) (Figure 3d).

#### PASI 90 response

Three RCTs with 893 patients reported the proportion of patients with PASI 90 response at weeks 24 and 52, respectively. There was no significant difference between secukinumab 300 mg and secukinumab 150 mg with respect to achieving a PASI 90 response at week 24 (OR=1.65, 95% CI=0.93-2.91, *p*=0.09), with slight heterogeneity between studies (I^2^= 62%) (Figure 2e). However, at week 52, secukinumab 300 mg was associated with a higher PASI 90 response rate than secukinumab 150 mg (OR=1.60, 95% CI=1.11-2.31, *p*=0.01), with no significant heterogeneity between studies (I^2^=0%) (Figure 3e).

### Dactylitis resolution and Enthesitis resolution

Three RCTs with 824 patients reported the resolution of dactylitis and enthesitis from baseline to 24 weeks. Secukinumab 300 mg was associated with a higher dactylitis resolution rate than secukinumab 150 mg (OR=1.42, 95% CI=1.06-1.91, *p*=0.02) (Figure 2f). However, there was no significant difference between secukinumab 300 mg and secukinumab 150 mg with respect to enthesitis resolution (OR=1.29, 95% CI, 0.99-1.68, *p*=0.06) (Figure 2g).

At week 52, we also assessed the resolution of dactylitis and enthesitis from baseline. There was no significant difference between secukinumab 300 mg and secukinumab 150 mg, regardless of dactylitis resolution (OR=1.07, 95% CI=0.67-1.71, *p*=0.78) (Figure 3f) or enthesitis resolution (OR=1.34, 95% CI=0.96-1.88, *p*=0.09) (Figure 3g).

### Safety

**Figure 4:**
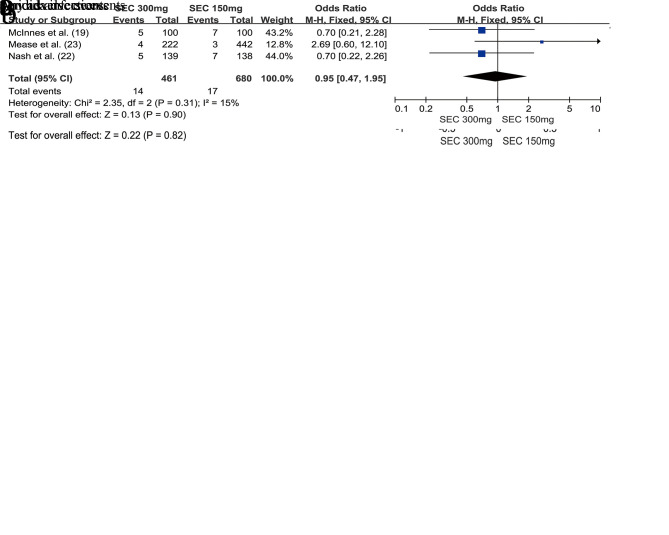
Forest plot depicting the safety of secukinumab 300 mg and secukinumab 150 mg in the treatment of patients with psoriatic arthritis at week 24.

**Figure 5:**
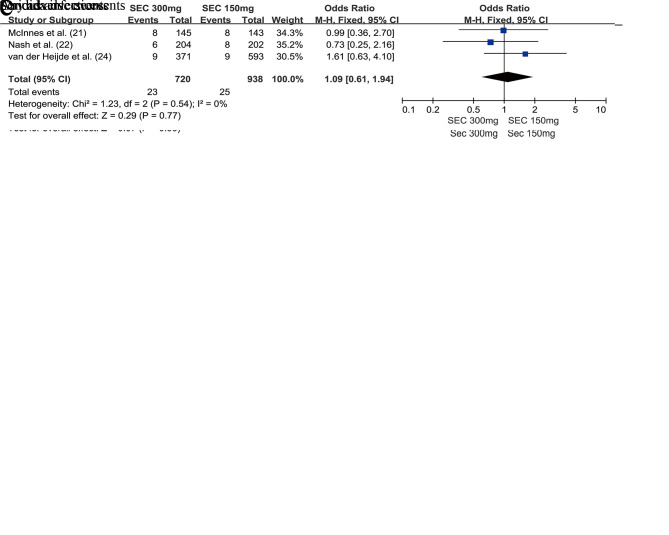
Forest plot depicting the safety of secukinumab 300 mg and secukinumab 150 mg in the treatment of patients with psoriatic arthritis at week 52.

We assessed the safety of the secukinumab 300 mg and secukinumab 150 mg groups at weeks 24 and 52, respectively. There was no significant difference between these groups with respect to the risk of any AEs (OR=0.98, 95% CI=0.77-1.25, *p*=0.88) (Figure 4a) and SAEs (OR=0.00, 95% CI=-0.02-0.02, *p*=0.82) at week 24 (Figure 4b). The same results were observed at week 52; we found that the secukinumab 300 mg group also was not at an increased risk of any AEs (OR=0.92, 95% CI=0.73-1.17, *p*=0.50) (Figure 5a) or SAEs (OR=1.01, 95% CI=0.72-1.42, *p*=0.74) (Figure 5b).

In addition, attention should be paid to the risk of developing Candida infections. There was no significant difference between the secukinumab 300 mg group and secukinumab 150 mg group with respect to the risk of developing Candida infections, whether at week 24 (OR=0.95, 95% CI=0.47-1.95, *p*=0.90) (Figure 4c) or at week 52 (OR=1.09, 95% CI=0.61-1.94, *p*=0.77) (Figure 5c).

## DISCUSSION

Our meta-analysis revealed that secukinumab 300 mg was more effective than secukinumab 150 mg at treating PsA patients without increasing the risk of developing any AEs or SAEs. At week 24, a significantly greater percentage of patients receiving secukinumab 300 mg (compared with those receiving secukinumab 150 mg) achieved ACR 20, ACR 50, PASI 75, dactylitis, and enthesitis resolution. At week 52, secukinumab 300 mg was also associated with a higher PASI 75 response rate and PASI 90 response rate than secukinumab 150 mg.

Subgroup analysis showed that secukinumab 300 mg had better efficacy than secukinumab 150 mg, especially in the anti-TNF-IR subgroup. At week 24, the advantages of secukinumab 300 mg (compared with secukinumab 150 mg) with respect to achieving ACR 20 and ACR 50 response were mainly concentrated in the anti-TNF-IR subgroup. At week 52, secukinumab 300 mg was also more effective than secukinumab 150 mg at achieving an ACR 20 response in the anti-TNF-IR subgroup.

Our meta-analysis suggests that secukinumab 300 mg is more beneficial to patients with PsA than secukinumab 150 mg in the short term without compromising safety, especially in PsA patients with anti-TNF-IR. Secukinumab 300 mg is also a good choice for long-term maintenance therapy in patients with PsA who have failed TNF inhibitors.

The number of circulating T helper-type 17 (Th17) cells increased significantly in RA patients after anti-TNFα therapy, suggesting that a Th17-targeted therapeutic approach may be beneficial for patients with anti-TNF-IR (26). This is also consistent with our results, in that a larger dose of secukinumab is required in patients with anti-TNF-IR.

IL-17A plays an important role in host defense against microorganisms and in the development of chronic inflammation (27,28). It has been reported that the rate of Candida infections with secukinumab treatment was higher than that with placebo (19,23,29). This is mostly related to the role of IL-17 in the mucocutaneous defense against Candida infections (30). All cases of Candida infection were resolved with standard oral treatment, and the patients continued to participate in this research. Safety analysis showed that secukinumab 300 mg did not increase the incidence of any AEs, SAEs, or Candida infections (compared to secukinumab 150 mg).

Comparison of the efficacy of secukinumab 300 mg and secukinumab 150 mg in PsA patients revealed that secukinumab 300 mg exhibits additional benefits at week 16 (31). However, secukinumab is not known to be effective in anti-TNF-naive and anti-TNF-IR subgroups; the same was observed in the safety analysis data for the long-term treatment of PsA patients. Our meta-analysis was able to compensate for the above deficiencies by not only comparing the efficacy in both anti-TNF-naive and anti-TNF-IR subgroups, but also for induction remission (24 weeks) and maintenance treatment (52 weeks). We defined ≤24 weeks as induction therapy and extended the observation endpoint to 52 weeks as maintenance therapy. We evaluated the difference in the efficacy and safety of secukinumab 300 mg and secukinumab 150 mg in different periods and subgroups. To the best of our knowledge, this has not been reported in a previous meta-analysis, and in addition, this is of great innovation in this field of research. Our study provides a basis for the selection of different doses of secukinumab in different groups of PsA patients during induction therapy and maintenance therapy.

However, our study has certain limitations. There were few data included in related studies, and no related indicators (such as PASI 75 response and PASI 90 response) for subgroup analysis. Secukinumab was administered at various regimens, with or without a loading dose. Further, the number of RCTs was limited. To acquire more accurate results, more high-quality, large-scale, long-term clinical trials are needed.

## CONCLUSION

In summary, secukinumab 300 mg was significantly more effective and well tolerated in both short-term induced remission and long-term maintenance therapy than secukinumab 150 mg, especially for PsA patients for whom TNF therapy has failed.

## AUTHOR CONTRIBUTIONS

Zhang KL and Wu D conceived and designed this study. Zhang KL and Wu D analyzed and interpreted the data. Zhang KL, Hou SY and Wu D contributed to data acquisition. All authors helped drafting the manuscript and its revisions for critically important intellectual content and approved the final manuscript version to be published.

## Figures and Tables

**Table 1:** Basic characteristics and risk bias of the included studies.

Trial	Dose and dosing schedule	No. of patients	Age (years)	Female, n (%)	Duration of psoriasis (years )	Weight (kg)	Treatment history of included patient	Modified Jadad Score	Study	Journal
FUTURE 2	SEC 300 mg: SEC 300 mg SC once a week from baseline to week 4 and then every 4 weeks	100	46.9±12.6	49 (49)	No data	85.4±18.4	Corticosteroids (≤10 mg/day PDN or equivalent) at a stable dose for ≥2 weeks; MTX ≤25 mg/week at a stable dose for ≥4 weeks; Anti-TNF-IR.	8	McInnes et al. (19)	Lancet
	SEC 150 mg: SEC 150 mg SC once a week from baseline to week 4 and then every 4 weeks	100	46.5±11.7	45 (45)	No data	91.2±19.8			Kavanaugh et al. (20) McInnes et al. (21)	J Rheumatol Rheumatology
FUTURE 3	SEC 300 mg: SEC 300 mg SC once a week from baseline to week 4 and then every 4 weeks	139	49.3±12.9	72 (51.8)	8.3±9.2	87.1±19.4	Corticosteroids (≤10 mg/day PDN or equivalent) at a stable dose for ≥2 weeks; MTX ≤25 mg/week at a stable dose for ≥4 weeks; Anti-TNF-IR.	8	Nash et al. (22)	Arthritis Research & Therapy
	SEC 150 mg: SEC 150 mg SC once a week from baseline to week 4 and then every 4 weeks	138	50.1±11.7	77 (55.8)	7.7±8.5	87.1±20.0				
FUTURE 5	SEC 300 mg with LD: SEC 300 mg SC once a week from baseline to week 3 and then every 4 weeks	222	48.9±12.8	114 (51.4)	6.7±8.3	81.9±16.9	Corticosteroids(≤10 mg/day PDN or equivalent), NSAIDs and MTX (≤25 mg/week) at a stable dose for the first 24 weeks of the study; Anti-TNF-IR.	8	Mease et al. (23)	Ann Rheum Dis
	SEC 150 mg with LD: SEC 150 mg SC once a week from baseline to week 3 and then every 4 weeks	220	48.4±12.9	109 (49.5)	6.7±7.1	83.3±19.6			van der Heijde et al. (24)	Rheumatology
	SEC 150 mg without LD: SEC 150 mg SC once a week from week 1 to week 3 and then every 4 weeks	222	48.8±11.8	102 (45.9)	6.2±6.1	84.1±20.5				

SEC, secukinumab; SC, subcutaneous; PDN, prednisone; MTX, methotrexate; LD: loading dose; Anti-TNF-IR: up to three anti-TNF agents could enroll if they had an inadequate response for at least 3 months or had stopped anti-TNF therapy because of safety/tolerability reasons.
